# Diabetes Foot Ulcer Prevention: A Review of Footwear Width Assessment for At‐Risk Feet

**DOI:** 10.1002/jfa2.70071

**Published:** 2025-08-03

**Authors:** Petra J. Jones, Alex V. Rowlands, Melanie J. Davies, Sicco A. Bus

**Affiliations:** ^1^ Leicester Diabetes Centre University Hospitals of Leicester Leicester General Hospital Leicester UK; ^2^ Diabetes Research Centre University of Leicester Leicester General Hospital Leicester UK; ^3^ NIHR Leicester Biomedical Research Centre University of Leicester Leicester UK; ^4^ Alliance for Research in Exercise Nutrition and Activity (ARENA) UniSA Allied Health and Human Performance Division of Health Sciences University of South Australia Adelaide Australia; ^5^ Amsterdam UMC University of Amsterdam Rehabilitation Medicine Amsterdam Movement Sciences Amsterdam the Netherlands

**Keywords:** assessment, diabetes, footwear, shoe size, shoes, width

## Abstract

**Background:**

Diabetes‐related foot ulceration (DFU) is often related to footwear fit. People with diabetes often have wider feet than in those without diabetes. Standards for evaluating footwear width in those at risk are therefore important.

**Methods:**

We performed a systematic search with a narrative review to assess consensus in quantitative methods used to assess footwear width for people at risk of DFU within research studies, and how often footwear is considered too narrow or wide. Search terms included diabet*, footwear, fit, size and width. This returned 1397 results, with 16 studies included after full paper review.

**Results:**

Three standards emerged, defining incorrectly fitting footwear as (1) one shoe size or (2) one width fitting larger or smaller than feet (4–7 mm) or (3) measured shoe width not equal to foot width. Footwear that was too narrow by one shoe size or width fitting was common (31.0%–78.0%) but too wide was rare (2 studies: 1.0% too wide where 100% had DM or 64.6% where 9% had DM). DFU was more likely in older people who wore either incorrect size or inappropriate footwear (*n* = 219, 100.0% DM, OR 1.7, *p* = 0.001) or incorrect length or width footwear (*n* = 440, 58.4% DM, OR 5.1, *p* = 0.02), but not in those with incorrect shoe width (*n* = 65, 9.0% DM, OR 0.75, *p* = 1.0).

**Conclusions:**

It is unclear how much space the at‐risk forefoot requires. Standardised methods are needed to establish the accuracy and reliability of foot and footwear measuring tools, and to evaluate footwear fit, given their relationship with the clinical outcome.

## 1 Introduction

Preventing diabetes‐related foot ulcers (DFU) is crucial as a history of ulceration doubles the risk of amputation [1], and in those who ulcerate, 5‐year mortality is around 30%–50% [2, 3, 4]. In addition to the substantial effect on quality of life [5], diabetes‐related foot disease is a global economic problem. Costs of ulcer treatment are substantial around the world [6, 7, 8, 9], with annual costs to the UK's National Health Service estimated at £1 billion even over a decade ago [10]. Treatment costs have continued to grow prompting comparisons of the direct costs of diabetes‐related foot disease to those of cancer [11].

Incorrectly fitting footwear can be a cause of foot ulcers in people with diabetes [12] and assessment of footwear fit is included in many international [13, 14] and national diabetes guidelines [15, 16, 17, 18]. Adequate footwear width arguably has a role to play in preventing diabetes‐related foot ulceration. We know that adherence to wearing footwear capable of reducing pressures below a pressure threshold (200 kPa) can reduce the risk of ulceration (relative risk −46%, odds ratio 0.38, *p* = 0.045) [19]. As the width fitting of shoes can have a significant impact on both in‐shoe forefoot plantar pressures [20, 21] and shear stress [21], ensuring adequate footwear width is likely also to reduce ulcer risk. Evaluating footwear width is also important as those with diabetes can have wider feet than those without [22, 23] and exceed standard footwear industry widths [24]. Measuring foot width is also important as foot width can also change even after people reach adulthood [25, 26], necessitating remeasurement. Inadequate width footwear has long been hypothesised to increase the risk of abrasion and lead to higher pressures or friction [27].

This review has three aims: (1) to describe how footwear width is quantitatively assessed in people with diabetes, that is, what replicable, objective methods that precisely distinguish correctly fitting from incorrectly fitting footwear are in use, (2) to summarise and compare methods of assessing diabetes footwear width, the rationale behind the methods and determine where there is consensus or divergence and (3) to summarise the percentage of people with diabetes wearing footwear of incorrect width and establish whether this increases the risk of foot ulceration.

## 2 Materials and Methods

### 2.1 Eligibility Criteria

**FIGURE 1:**
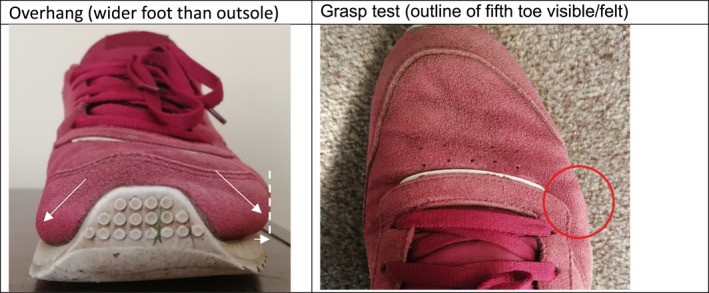
Subjective assessment of diabetes footwear width.

Inclusion criteria were assessments of footwear worn (i) by a cohort that included people with diabetes and (ii) with quantitative measurement either of foot width compared with habitual or retail footwear width or measurement of width of both feet and footwear. Studies involving subjective assessments of footwear width which did not involve any measurement were excluded. The subjective assessment of footwear width can include the visual inspection of the shoe upper material [28, 29], the tightness of lacing [30], or presence of ‘overhang’, that is, a foot that is wider than the outsole while the shoe is worn [31] (Figure 1). Other examples include tactile palpation of the shoe to determine adequate width, such as the ‘grasp test’ [32].

It has been suggested that the grasp test can have inconsistent results with poor intra‐ and inter‐rater reliability observed over time [32]. The disadvantages of subjectively assessing footwear width also include the challenge of passing such skills on to new starters and ensuring consistency among healthcare professionals who utilise them.

### 2.2 Search Strategy

We carried out a systematic search of Scopus, Google Scholar (any date until 02/12/2024) and Medline (any date until 02/12/2024) databases. On Scopus, the search query searched within all fields of the records. For Medline and Google Scholar, this option was not available and therefore the query considered only keywords. We did not set limits on the publication date. All peer‐reviewed studies summarised in English language were considered eligible for inclusion, irrespective of the year of publication.

Search terms included diabet*’ AND ‘footwear’ AND ‘fit’ AND ‘width’, ‘diabet*’ AND ‘shoe’ AND ‘fit’ AND ‘width’ and, finally, diabet* AND (‘shoe’ OR ‘footwear’) AND ‘width on all three databases.

An additional search of diabet* AND (‘shoe size’ OR ‘footwear size’) was carried out on all three databases (any date until 02/12/2024).

## 3 Results

### 3.1 Methods of Measuring Width of Feet and Footwear of People With Diabetes

**FIGURE 2:**
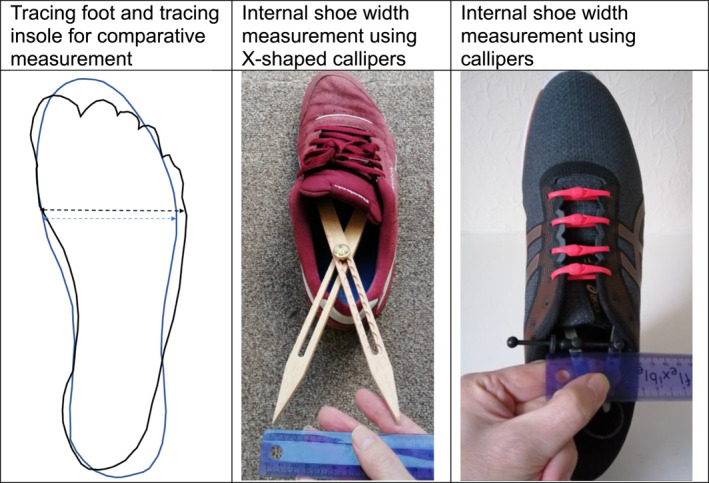
Objective assessment of diabetes footwear width.

The search produced 1397 results after the removal of duplicates of which 16 studies met the inclusion criteria, outlined in Table 1. The objective assessment of footwear width typically involves comparative measurement: for example, measuring both foot and footwear width and comparing the two (*n* = 12) [34, 35, 36, 38, 39, 40, 41, 42, 43, 44, 46, 47] or alternatively measuring feet and comparing with industry‐reported (*n* = 1) [24] or individual‐reported (*n* = 2) [37, 45] or measured shoe size (*n* = 1) [33]. Methods include tracing feet and footwear (*n* = 2) [43, 47], using a ruler to compare measurements (Figure 2). Alternatively, both feet and footwear are measured using callipers (*n* = 4) [36, 38, 39, 40], slide rules (*n* = 3) such as Brannock devices [41], or Ritz sticks [34, 42] or a combination of devices (*n* = 3) [35, 44, 46] including rulers [35] or tape measures [46] (Table 1). Remaining studies measured feet or shoes only with similar devices (*n* = 4) [24, 33, 37, 45].

**TABLE 1:** Consistency of footwear fit width standards in studies involving people with diabetes.

Lead author	Year	Size	% DM		Country	Measurement method	
						Shoe	Feet
Incorrect fit (standard 1 shoe size)							
Meyr [33]	2011	129	100.0%	1 shoe size smaller	US	—	Brannock
							Ritz stick^b^
Nixon [34]	2006	440	58.4%	1 shoe size smaller/larger^a^	US	Ritz stick	Ritz stick
Obimbo [35]	2008	219	100.0%	1 shoe size smaller^a^	Kenya	Ruler	Ritz stick
Pataky [36]	2007	426	22.5%	1 shoe size smaller/larger	Switzerland	Callipers	Callipers
Schwarzkopf [37]	2011	235	18.3%	½ shoe size smaller/larger	US	N/A	Clarks metre
Incorrect fit (standard 2 width fitting)							
Burns [38]	2002	65	9.0%	> 7 mm difference between shoe and foot (> 1 UK width fitting)	UK	Callipers	Callipers
Chaiwanichsiri [39]	2008	213	M: 23%	> 5 mm difference between shoe (i.e., ± 5 mm) and foot	Thailand	Callipers (sliding)	Callipers (X‐shape)
			F: 17%				
Harrison [40]	2007	100	100.0%	> 7 mm difference between shoe and foot (> 1 UK width fitting, ± 7 mm)	UK	Callipers	Callipers
Lee [41]	2004	165	100.0%	≤ 4 mm smaller shoe than foot	S. Korea	Brannock	Brannock
Reddy [42]	1989	70	100.0%	1 width fitting larger	UK	Ritz stick	Ritz stick
		50	0.0%				
Reveal [43]	2001	100	100.0%	> 6 mm smaller shoe than foot	US	Trace	Trace
Incorrect fit (standard 3 Shoe = Foot)							
Chantelau [24]	2002	568	100.0%	Shoe size < foot width	Germany	WMS devices	Industrial shoe size
		100	0.0%				
Isip [44]	2016	78	100.0%	Shoe size ≠ foot width	Philippines	Brannock	Callipers
Paiva De Castro [45]	2010	399	18.0%	Shoe size ≠ foot width	Brazil	N/A	Callipers
Tsuruoka [46]	2020	30	100.0%	Shoe size < foot width	Japan	Brannock	Tape measure
		30	0.0%				
Woldemariam [47]	2020	161	100.0%	Shoe size ≤ foot width + reddened areas of foot	Ethiopia	Trace	Trace

*Note:* Year, year paper published; size: size of cohort; %DM, % with diabetes mellitus where 0.0% indicates a control group; incorrectly fitting, criteria for incorrectly fitting footwear where mm is the permissible difference between the shoe and foot width specified in millimetres; Country: UK, United Kingdom; US, United States; N/A, not applicable; —, unreported.

^a^ Determined through correspondence with the author.

^b^Both devices were used to measure feet.

The accuracy or reliability of these devices or the relative merits of each device are rarely discussed in assessments of footwear worn by people with diabetes—only the reliability of the custom callipers used in Chaiwanichsiri [39] was explicitly evaluated in a prior study (ICC 0.985 (95% CI 0.972–0.992)) [48]. Information on the accuracy and reliability of foot and footwear measuring tools is scarce even outside diabetes foot and footwear measurement (see Supporting Information S1: Table S1).

### 3.2 Standards for Assessing Adequate Width in Footwear Worn by People With Diabetes

Although standards for adequate footwear width vary, three standards for diabetes footwear width were used in the studies in our review where incorrectly fitting footwear (1) differs by a shoe size from feet, (2) differs by a width fitting from feet or (3) where footwear width measurement differs from foot width. The first standard is based on shoe size (*n* = 6 studies): shoes which are one shoe size smaller [33, 35], larger [42] or smaller or larger than a person's measured foot width are incorrectly fitting [34, 36] (Table 1). Sometimes this can be a half size smaller or larger than the measured foot width [37]. This is complicated by variations in shoe size from country to country and even sometimes between shoe manufacturers. These studies were carried out in different countries including the United States [33, 34], UK [42], Kenya [35] and Switzerland [36].

**FIGURE 3:**
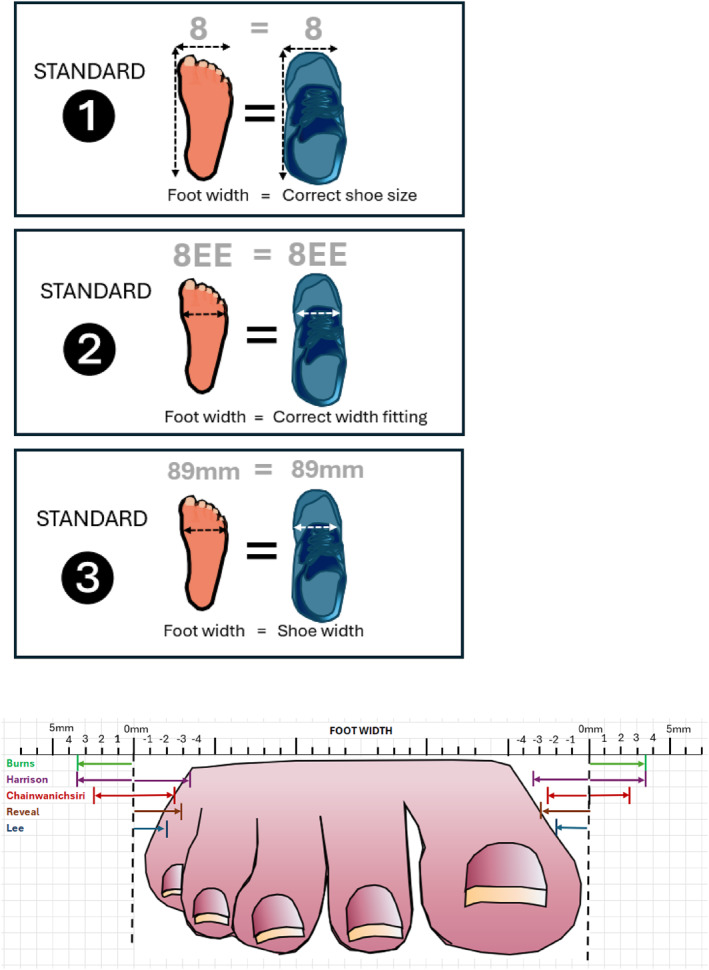
Three standards of diabetes footwear width.

Within peer‐reviewed studies involving the objective assessment of footwear width worn by people with diabetes, the second standard for assessing foot width is based on width fitting. Width fittings differ from shoe sizes in that a shoe size 7 has both a smaller length and width to a size 8, whereas a shoe size 8E has only a smaller width dimension than a shoe size 8EE (Figure 3). If the foot and shoe width differ by one width fitting or more, they are deemed incorrectly fitting [38, 40, 42]—this is equivalent to up to a 7‐mm difference between the foot and footwear.

In other studies, a width fitting difference between the foot and shoe ranged from 4 mm [41] to 6 mm [43], presumably influenced by width fittings within those countries (South Korea [41], Thailand [39] and the United States [43], see Table 1). Width fittings can differ between retailers and use various classifications, for example, E, EE, EEE, 4E; E, F, G, H etc., that are potentially confusing to people with diabetes.

Specifying ranges, therefore, for acceptable differences between foot and shoe width (see Figure 3, e.g., in millimetres, centimetres or inches) is important both for people with diabetes unsure how much difference is acceptable and healthcare professionals or researchers evaluating footwear width.

Finally, the third standard was for shoe width as measured in centimetres or millimetres to be equal to foot width [44, 45, 46, 47] where incorrectly fitting footwear had a narrower width than the foot [46] or where footwear was equal to foot width but reddened areas of the foot were discovered on inspection [47]. The issue here is that this standard gives no range for acceptable differences between foot and shoe width, leaving some ambiguity for those assessing appropriate width.

Isip et al. [44] is the only study to expressly adopt the footwear width standard from the IWGDF guidelines [14], namely that the internal width of the shoe should be equal to the foot width (analogous to Standard 3). In contrast, although Tsuruoka et al. [46] and Woldemariam et al. [47] are both aware of the IWGDF guidelines (cited within their papers), they suggest their own footwear width standard without further explanation, either suggesting that footwear width should be equal to or larger than foot width [46] or wider than foot width [47]. The remaining studies predate (1989–2011) the IWGDF guidelines and the rationale behind their thresholds is rarely discussed. Possible explanations such as the need to accommodate changes in foot width during particular phases of gait, oedema‐related fluctuations in foot width or swelling related to sustained physical activity are not pursued and this lack of discussion inhibits debate.

### 3.3 Prevalence of Incorrectly Fitting Footwear Worn by People With Diabetes and Evidence of a Relationship With Risk of Ulceration

Of the 16 studies included in our review, 10 included cohorts where 100% of the participants had diabetes [24, 34, 36, 38, 42, 43, 44, 45, 47, 48] (Table 1).

The percentage of people with diabetes wearing footwear that is one size or more larger or smaller (Standard 1) is 37.1% [42] to 78.0% [33], whereas those wearing footwear of correct length but incorrect width are unreported. About 37.1% [42] to 76.0% [40] of participants wore footwear that was one or more width fitting larger or smaller than their feet and therefore incorrectly fitting (Standard 2, based on four studies [40, 41, 42, 46]). Where reported, typically footwear was too narrow by one width fitting in more than one third of people with diabetes in each study (*n* = 4 studies [39, 40, 41, 43]; Table 2). A width fitting difference varied from 4 mm (0.4 cm) [41] to 7 mm [40]. Only two studies reported footwear being worn that was too wide [38, 40]. In a study comprised only of people with diabetes (*n* = 100), just 1.0% of the cohorts were wearing footwear one width fitting too wide (7 mm) [40], whereas in a study of older people more generally (*n* = 65) that included 9% with diabetes, 64.6% wore footwear that was one width fitting too wide (7 mm) [38] (Table 2).

**TABLE 2:** Prevalence of diabetes footwear worn with incorrect width.

Lead author	Study size (% DM)		% Cohort with incorrect footwear fit			
			Incorrect width	Too wide	Too narrow	Incorrect length or width
Standard 1 correct shoe size						
Meyr [33]	129 (100.0%)	1 shoe size smaller than foot	—	—	—	78.0%
Nixon [34]	440 (58.4%)	1 shoe size smaller/larger than foot	—	—	—	74.5%^a^
Obimbo [35]	219 (100.0%)	1 shoe size smaller than foot	—^f^	—	—	—
Pataky [36]	426 (22.5%)	1 shoe size smaller/larger than foot	—	—	—	91.3%^a^
Schwarzkopf [37]	235 (18.0%)	½ shoe size smaller/larger than foot	—	—	—	44.2%^h^
Standard 2 correct width fitting						
Burns [38]	65 (9.0%)	> 7 mm difference between shoe and foot (> 1 UK width fitting)	70.8%^a^	64.6%^a^	6.2%^a^	72.3%^a^
Chaiwanichsiri [39]	213 (17.1%–20.3%)	Shoe width = foot width + 5 mm max	—	—	34.3%–50.0%^b^	—
Harrison [40]	100 (100.0%)	Shoe width = foot width + 7 mm max	44%–47%^d^	1.0%	43.0%–46.0%^d^	76.0%
Lee [41]	165 (100.0%)	Shoe width 4 mm ≤ foot width	36.4%	—	36.4%	—
Reddy [42]	70 (100.0%)	1 width fitting smaller/larger than foot	—	—	—	37.1%
	50 (0.0%)					24.0%
Reveal [43]	100 (100.0%)	Shoe width 6 mm < foot width	—	—	31.0%	—
Standard 3 foot width = Shoe width						
Chantelau [24]	568 (100.0%)	Shoe width = foot width	—	—	66.0%^c^	—
	100 (0.0%)					
Isip [44]	78 (100.0%)	Shoe width = foot width	37.2%^e^	—	—	—
Paiva De Castro [45]	399 (18.0%)	Shoe width = foot width	—^g^	—	—	—
Tsuruoka [46]	30 (100.0%)	Shoe width < foot width	—	—	—	46.7%^i^
	30 (0.0%)					80.0%^i^
Woldemariam [47]	161 (100.0%)	Shoe size = foot width with reddened areas of foot	—	—	—	—^j^

^a^ Percentage of cohorts wearing incorrectly fitting footwear includes both people with and without diabetes. In Burns [38], the percentage of people with diabetes wearing incorrectly fitting footwear was 100% (*n* = 6). %DM = % with diabetes mellitus where 0.0% indicates a control group; size: size of cohort; — unreported. None of the six people with diabetes were wearing correctly fitting footwear for width.

^b^ Diabetes melllitus: 34.3% of males and 50.0% of females with diabetes wore too narrow footwear.

^c^ Reported as ‘more than two‐thirds’.

^d^ Separate percentages reported for left and right feet.

^e^ Based on 29 of 78 participants whose footwear was measured.

^f^ No breakdown of ‘risky footwear’ which includes both inappropriate and incorrectly fitting footwear.

^g^ Results for incorrect length individual sizes only reported.

^h^ Percentage reported refers to people with diabetes.

^i^ Calculated by deducting percentage of correctly fitting from 100%.

^j^ Percentage of well and ill fitting reported do not add up to 100%, likely errors.

When it was assessed whether measured foot width and footwear width were equal (Standard 3, International Working Group on Diabetic Foot guidelines), again a third of people with diabetes were found wearing footwear of incorrect width (37.2%) but this was based on only one study [44] and unreported in other studies [45, 46, 47]. Further detail on the percentages wearing footwear that was too wide or too narrow was not provided.

The remaining six studies assessed footwear width in older people (average age 68.7%–82.0%) where 9.0 [38] to 50.0% [39] of people had diabetes (DM) [24, 36, 37, 38, 39, 45], older veterans (average age 67.2, 58.4% DM [34]) or three populations within New York City (average age 52, 18.3% DM [37], Supporting Information S1: Table S2). Among studies involving older people which did not report percentages of people with diabetes wearing footwear of incorrect width, 72.3 [38] to 91.3% [36] wore incorrectly fitting footwear.

Only three of the 16 included studies assessed the relationship between footwear fit and diabetes‐related foot ulceration (Table 3). In Burns et al., while incorrect footwear length increased the risk of ulceration 10 times (odds ratio 10.0, *p* = 0.016), incorrect footwear width was not statistically significant (odds ratio 0.75, *p* = 1) [38]. However, people with diabetes accounted for just 9.0% of this small cohort (*n* = 65). In a larger study (*n* = 440) by Nixon et al. [34] where people with diabetes made up 58.4% of the cohort, those wearing incorrect footwear length or width were five times more likely to ulcerate (odds ratio 5.1, *p* = 0.02); however, a separate statistical analysis for incorrect footwear width as distinct from the incorrect footwear length was not provided. Finally, in Obimbo et al. [35] (*n* = 219), risky footwear (footwear that was either a shoe size too small or inappropriate such as flip flops or sandals) increased the risk of ulceration (odds ratio 1.7, *p* = 0.001) but did not provide separate statistical analysis for the effect of incorrect fit upon ulceration as distinct from inappropriate footwear (Table 3).

**TABLE 3:** Factors influencing poor footwear fit and relationship with diabetes‐foot ulceration (DFU).

Lead author	% DM	Total	Relationship between footwear fit and DFU	Factors‐related incorrect width
Standard 1 correct shoe size				
Meyr [33]	100.0%	129	Not applicable	Not assessed
Nixon [34]	58.4%	440	*Incorrect footwear length or width*:	Not assessed
			Odds ratio 5.1, *p* = 0.02	
Obimbo [35]	100.0%	219	*Risky footwear (size too small or flip flops/sandals used)*:	Not assessed
			Odds ratio 1.7, *p* = 0.001	
Pataky [36]	22.5%	426	Not applicable	Not assessed
Schwarzkopf [37]	18.3%	235	Not applicable	*Male gender*:
				Odds ratio 2.2, *p* = 0.02
Standard 2 correct width fitting				
Burns [38]	9.0%	65	*Incorrect footwear length and DFU*:	Incorrect footwear length associated with sensory impairment (*p* = 0.03)
			Odds ratio 10.0, *p* = 0.016	
			*Incorrect footwear width and DFU*:	
			Odds Ratio 0.75, *p* = 1 (not significant)	
Chaiwanichsiri [39]	M:24.0%	213	Not applicable	Not assessed
	F: 50.0%			
Harrison [40]	100.0%	100	Not applicable	
Lee [41]	100.0%	165	Not applicable	Not assessed
Reddy [42]	100.0%	70	37% DM versus 24% general patients (*p* = 0.05)	Not assessed
	0.0%	50		
Reveal [43]	100.0%	100	Not applicable	No statistical significance reported
Standard 3 foot width = Shoe width				
Chantelau [24]	100.0%	568	Not applicable	Not assessed
Isip [44]	100.0%	78	Not applicable	Not assessed
Paiva De Castro [45]	18.0%	399	Not applicable	Not assessed
Tsuruoka [46]	100.0%	30	Not applicable	Not assessed
	0.0%	30		
Woldemariam [47]	100.0%	161	Not applicable	Not assessed

Only three of these studies assessed why people with diabetes were wearing footwear with incorrect length or width. In Burns et al. [38], incorrect footwear length was associated with sensory impairment (*p* = 0.03) but presumably not footwear width which was unreported. In Reveal et al. [43], only 12% of the cohort had received footwear education, but the statistical significance of its association with wearing incorrectly fitting footwear was unreported. In Schwarzkopf et al. [37], male members of the cohort were twice as likely to be wearing footwear that did not fit (odds ratio 2.2, *p* = 0.02), but no explanation as to why was given (Table 3).

## 4 Discussion

Only one of the studies [44] examining footwear width referred to national or international guidance on footwear fit (Supporting Information S1: Table S3)—in this instance, the International Working Group on Diabetic Foot practical guidelines [13], suggesting that many researchers either are not aware of these standards or choose not to adopt them for some reason. The extent to which the scarcity of formal studies examining the accuracy and reliability of traditional foot and footwear measuring tools (see Supporting Information S1: Table S1) has contributed to this is unclear. There is a need for the accuracy, ease of use, inter‐ and intra‐rater reliability of these foot and footwear measuring tools to be formally evaluated. It is worth noting that in some communities, flip flops, sandals or other forms of footwear may be worn extensively depending on culture, climate, available income, health literacy or occupation, which might limit the application of guidelines in their current form, requiring pragmatic strategies to reduce ulcer risk in this context [49].

An ongoing challenge is that the vast majority of these studies provided no explanation of the reasoning behind footwear width standards or even a citation to a prior study or discussion with the exception of an industrial standard for shoe sizing used in one study [24]. Even less attention was paid to heel width with assessments performed in just two of these studies ([40, 46] see Supporting Information S1: Table S4).

Studies assessing adequacy of footwear length easily outnumber those assessing width, with many omitting to report on footwear width entirely. It is unclear whether this is due to some unconscious bias that insufficient footwear length is more likely to lead to ulceration than insufficient width.

Strengths of this review include its systematic approach to searching three key databases for literature (Scopus, Google Scholar and Medline) and its narrative format provides a scope to explore the many issues raised by current approaches. However, we acknowledge its limitations given the abundance of descriptive terms that may be used in this context: for example, the use of breadth rather than width, studies which refer to ‘size of shoe’ without mentioning either breadth or width and so on. To the authors' knowledge, this is the first review to specifically focus upon summarising methods for measuring width in footwear worn by people with diabetes. However, key questions remain—particularly how much space within a shoe a foot biomechanically needs at the metatarsal heads during the various phases of gait. Only a handful of studies have been carried out [50, 51], with only one small study (*n* = 20) that involved people with diabetes [52] and focused on the effect of walking speed on foot deformation rather than the dynamic changes in foot width between standing and walking. This is a fundamental question to answer which would inform footwear width standards for those with diabetes. Finally, the efficacy of adhering to these alternate footwear width standards in reducing diabetes‐related ulcer risk has yet to be compared.

## 5 Conclusion

Three standards emerge within research studies for assessing the width of footwear worn by people with diabetes (based on foot width equalling shoe width or shoes within one size or width fitting of feet) within research studies. However, the rationale behind these standards is rarely explained and national/international guidelines for footwear fit are rarely adopted. Based on these standards, 31.0%–78.0% of people with diabetes wear footwear that is too narrow (either by one shoe size or width fitting of 4–7 mm). There has yet to be a large study (*n* ≥ 100) analysing the relationship between adequacy of footwear width and diabetes foot ulceration. Further research is required to understand biomechanically how much space the at‐risk forefoot requires with much necessary groundwork establishing the accuracy and reliability of foot and footwear measuring tools yet to be done.

## Author Contributions

**Petra J. Jones:** conceptualization, formal analysis, investigation, methodology, visualization, writing – review and editing. **Alex V. Rowlands:** writing – review and editing. **Melanie J. Davies:** writing – review and editing. **Sicco A. Bus:** writing – review and editing.

## Ethics Statement

The authors have nothing to report.

## Consent

The authors have nothing to report.

## Conflicts of Interest

The authors declare no conflicts of interest.

## Supporting information

Supporting Information S1

Supporting Information S2

## Data Availability

The data that support the findings of this study are available from the corresponding author upon reasonable request.
