# Supplementary material for: Diabetes Foot Ulcer Prevention: A Review of Footwear Width Assessment for At‐Risk Feet
Source: J Foot Ankle Res. 2025 Aug 3;18(3):e70071. doi: 10.1002/jfa2.70071 (PMC12318611; doi:10.1002/jfa2.70071)
Supplement: Supplementary file 1 — Supporting Information S1 [file JFA2-18-e70071-s002.docx]

**Supplemental materials

SUPP. TABLE 1 Reliability of foot and footwear width measuring devices**

| **Foot width measuring devices** | | | | | | | | |
| --- | --- | --- | --- | --- | --- | --- | --- | --- |
| **Measuring device** | **Accuracy  (error margin in mm)** | | **Reliability** | | | | | |
|  |  |  | **Intraclass** | | **Interclass** | | **Test-Retest** | |
|  | Width | Length | Width | Length | Width | Length | Width | Length |
| Brannock device [S1] | ± 4 mm | |  |  | -- | -- | -- | -- |
| Callipers [S2] | -- | -- | 0.98 (0.98-0.99) | 1.00 (0.99-1.00) | 0.94  (0.86-0.97) | 0.85  (0.75-0.92) | -- | -- |
| Callipers [S3] | ±1-2mm | -- | (0.87-0.99) |  | -- | -- | -- | -- |
| Ritz stick | -- | -- | -- | -- | -- | -- | -- | -- |
| Ruler | -- | -- | -- | -- | -- | -- | -- | -- |
| Tape measure [S4] | ± 2 mm | ± 2 mm | -- | -- | -- | -- | -- | -- |
| Tape measure with stick [S5] | -- | ± 2 mm | -- | -- | -- | -- | -- | -- |
| Tracing [S6] | -- | -- | -- | -- | -- | -- | 0.85  (0.72–0.93) | 0.98  (0.95–0.99) |
| **Footwear width measuring devices** | | | | | | | | |
| **Measuring device** | **Accuracy  (error margin in mm)** | | **Reliability** | | | | | |
|  |  |  | **Intraclass** | | **Interclass** | | **Test-Retest** | |
|  | Width | Length | Width | Length | Width | Length | Width | Length |
| Brannock | -- | -- | -- | -- | -- | -- | -- | -- |
| Callipers | -- | -- | -- | -- | -- | -- | -- | -- |
| Ritz stick | -- | -- | -- | -- | -- | -- | -- | -- |
| Trace | -- | -- | -- | -- | -- | -- | -- | -- |

**KEY**mm = Millimetres; -- = Unreported. Accuracy: Error margin shown as plus or minus in millimetres (mm).
Whilst 0.96-0.99 is sometimes given as the intraclass correlation coefficient of reliability for the Brannock device, this is actually in relation to measuring foot volume rather than individual foot length or width [S7].

**References for SUPP. TABLE 1**

[S1] Amini M, Vasefi F, MacKinnon N, Validation of hand and foot anatomical feature measurements from smartphone images. Imaging, Manipulation, and Analysis of Biomolecules, Cells, and Tissues 2018; XVI; 1049711. <https://doi.org/10.1117/12.2288026>.
[S2] Janchai S, Tantisiriwat N, Reliability of caliper. J. Medical Assoc. Thailand 2005; 88(Suppl 4):S85–S89.
[S3] Ballester A., Piérola A., Parrilla E., Nácher B., (2017). Fast, portable and low-cost 3D foot digitzers: Validity and reliability of measurements. Eighth international conference and exhibition on 3D body scanning and processing technologies, Montreal, Canada, 11-12 October 2017. <http://dx.doi.org/10.15221/17.218>.
[S4] Tang UH, Siegenthaler J, Hagberg K, Karlsson J, Tranberg R, Foot anthropometrics in individuals with diabetes compared with the general Swedish population: Implications for shoe design. Foot & Ankle 2017; 10(3):1. DOI:10.3827/faoj.2017.1003.000.
[S5] Armitage Z, Foot scanning in UK, USA and China. Proceedings of 3DBODY.TECH 2018 9th Int. Conference and Exhibition on 3D Body Scanning and Processing Technologies, Lugano, Switzerland, 16-17 Oct 2018, pp. 275–280, <https://doi.org/10.15221/18.275>.
[S6] Menz HB, Tiedemann A, Mun-San Kwan M, Latt MD, Sherrington C, Lord SR, Reliability of clinical tests of foot and ankle characteristics in older people. J Am Podiatr Med Assoc 2003; 93(5): 380–387. <https://doi.org/10.7547/87507315-93-5-380>.
[S7] Friends J, Augustine E, Danoff J, A comparison of different assessment techniques for measuring foot and ankle volume. JAPMA 2008; 98(2): 85–94.
<https://doi.org/10.7547/0980085>

*Supp. Table S1 search criteria*

("accuracy" OR "accurate") AND ("shoe" OR "footwear") AND ("measuring stick" OR "foot measure") Google Scholar

**SUPP. TABLE S2 Demographics in diabetes footwear width assessment studies**

| **Lead author** | **%DM** | **Study Size (n)** | **% cohort incorrect  length or width** | **% cohort incorrect  width** | **% neuropathy** | **Neuropathy threshold** | **%Cohort =Female** | **Age (Years)** |
| --- | --- | --- | --- | --- | --- | --- | --- | --- |
| Burns [38] | 9.0% | 65 | 72.3%* | 70.8%* | 26.0% | M/F 6/8 sites | 60.0% | 82.0● (64-93) |
| Chaiwanichsiri [39] | M:24.0% F: 50.0% | 213 | -- | -- | -- | -- | 49.3% | 68.7 ± 5.4 |
| Chantelau [24]^1^ | 100.0% | 568 100 | -- | -- | 100.0% 0.0% | -- | -- 44.0% | 64.0 |
| Harrison [40] | 100.0% | 100 | 76.0% | 44-47% | 20.0% | M/F 8/10 sites | 48.0% | 62.0 ± 14.9 |
| Isip [44] | 100.0% | 78 | -- | 37.2% | 7.1% | MF 2/3 apps @ 8 sites | 72.9% | 63.9 ± 9.4 |
| Lee [41] | 100.0% | 165 | -- | 36.4% | 47.3% | M/F1 of 4 sites | -- | 59.3 (39-90) |
| Meyr [33] | 100.0% | 129 | 78.0% | -- | -- | -- | 60.5% | 62.2 |
| Nixon [34] | 58.4% | 440 | 74.5%* | -- | -- | -- | 5.9% | 67.2 ± 12.5 |
| Obimbo [35] | 100.0% | 219 | -- | -- | 90.2% | -- | 54.8% | -- |
| Paiva De Castro [45] | 18.0% | 399 | -- | -- | -- | -- | 56.9% | 69.6 ± 6.8 |
| Pataky [36] | 22.5% | 436 | 91.3%* | -- | 25.8% | Tuning fork | 56.6% | 76.3 ● 14.1 |
| Reddy [42] | 100.0% 0.0% | 70 50 | 37.0% 24.0% | -- | 31.4% | Vibration @ ankles | 45.7% 44.0% | 52 (14-70) 49 (28-72) |
| Reveal [43] | 100.0% | 100 | -- | -- | 20.0% | M/F n/7 sites | 53.0% | 52.0 |
| Schwarzkopf [37] | 18.3% | 235 | 44.2% (DM) | -- | -- | -- | 28.6% | 46.5 (18-82) |
| Tsuruoka [46] | 100.0% 0.0% | 30 30 | 46.7% 80.0% | -- | 100.0% 46.7% | M/F 2/n sites | 46.7% 46.7% | 71● (67-79) 72● (66-79) |
| Woldemariam [47] | 100.0% | 161 | -- | -- | -- | -- | 47.2% | 50.6 ± 6.3^2^ 51.5 ± 16.6 |

**KEY**
Neuropathy threshold: M/F = Monofilament test followed by threshold number of sites to constitute loss of sensation associated with neuropathy. Apps refers to applications of the monofilament. N indicates an unspecified number of sites or locations. Age: Mean and standard deviation unless specified. ● Indicates median followed by range. ^1^ Neuropathy paper cited but still unclear which tests/thresholds applied. ^2^ Demographics broken down into groups of ulcer cases and controls.

**SUPP. TABLE S3 National/international standards and other sources of
 recommended footwear fit**

| **Lead author** | **Incorrect footwear width definition** | **Type of standard e.g. guideline, study, article, book** | **Lead author of cited source (if applicable)** |
| --- | --- | --- | --- |
| Burns [38] | A half British shoe size OR one British width fitting (7mm) | Unspecified | Not applicable |
| Chaiwanichsiri [39] | >5mm difference between shoe (i.e. ±5mm) & foot | Unspecified | Not applicable |
| Chantelau [24] | Foot width equal to industrial standard shoe widths | Industrial standard shoe sizes | Fagus, Alfeld/Leine Citation not provided |
| Harrison [40] | > one British width fitting difference | Unspecified | Not applicable |
| Isip [44] | Equal to foot width @ metatarsal joints | Guideline - IWGDF | Bakker et al. [R1] |
| Lee [41] | Footwear must be equal to foot + ≥ 5mm | Unspecified | Not applicable |
| Meyr [33] | 1 shoe size smaller | Unspecified | Not applicable |
| Nixon [34] | 1 shoe size smaller/larger | Unspecified | Not applicable |
| Obimbo [35] | 1 shoe size smaller | Unspecified | Not applicable |
| Paiva De Castro [45] | Shoe size ≠ Foot width | Unspecified | Not applicable |
| Pataky [36] | 1 shoe size smaller/larger | Unspecified | Not applicable |
| Reddy [42] | ≥ one British width fitting (loose or tight) | Unspecified | Not applicable |
| Reveal [43] | Shoe width 6mm < foot width | Unspecified | Not applicable |
| Schwarzkopf [37] | ½ shoe size smaller/larger | Unspecified | Not applicable |
| Tsuruoka [46] | Shoe size < Foot width | Unspecified | Not applicable |
| Woldemariam [47] | Shoe size ≤ Foot width +  reddened areas of foot | Unspecified | Not applicable |

**Table S5 References**

[R1] Bakker K, Appelqvist J, Schaper J, for the International Working Group on the Diabetic Foot Editorial Boardt. Practical guidelines on the management and prevention of the diabetic foot 2011. Diabetes Metab Res Rev. 2012;28(Suppl 1):225–31. http://dx.doi.org/10.1002/ dmrr.2253.

**SUPP. TABLE S4 Heel width assessment in diabetes footwear**

| **Lead author** | **% DM** | **Heel width measured?** | **Shoe heel width assessed?** |
| --- | --- | --- | --- |
| Burns [38] | 9.0% | NO | NO |
| Chaiwanichsiri [39] | M:20.3% F: 17.1% | YES | NO |
| Chantelau [24] | 100.0% | NO | NO |
| Harrison [40] | 100.0% | NO | NO |
| Isip [44] | 100.0% | NO | NO |
| Lee [41] | 100.0% | NO | NO |
| Meyr [33] | 100.0% | NO | NO |
| Nixon [34] | 58.4% | NO | NO |
| Obimbo [35] | 100.0% | NO | NO |
| Paiva De Castro [45] | 18.0% | YES | NO |
| Pataky [36] | 22.5% | NO | NO |
| Reddy [42] | 100.0% 0.0% | NO | NO |
| Reveal [43] | 100.0% | NO | NO |
| Schwarzkopf [37] | 18.3% | NO | NO |
| Tsuruoka [46] | 100.0% 0.0% | NO | NO |
| Woldemariam [47] | 100.0% | NO | NO |

**SUPP. TABLE S5 Percentage of female study participants calculations**

|  | **All participants** | | | |  |
| --- | --- | --- | --- | --- | --- |
| **Lead author** | **Male** | **Female** | **Total** | **%Female** | **%Male** |
| Burns [38] | 26 | 39 | 65 | 60.0% | 40.0% |
| Chaiwanichsiri [39] | 108 | 105 | 213 | 49.3% | 50.7% |
| Chantelau [24] | -- 56 | -- 44 | 568 100 | -- 44.0% | -- 56.0% |
| Harrison [40] | 52 | 48 | 100 | 48.0% | 52.0% |
| Isip [44] | -- | -- | 170* 78* | 72.9% | 27.1% |
| Lee [41] | -- | -- | -- | -- | -- |
| Meyr [33] | 51 | 78 | 129 | 60.5% | 39.5% |
| Nixon [34] | 414 | 26 | 440 | 5.9% | 94.1% |
| Obimbo [35] | 99 | 120 | 219 | 54.8% | 45.2% |
| Paiva De Castro [45] | 172 | 227 | 399 | 56.9% | 43.1% |
| Pataky [36] | 185 | 241 | 426 | 56.6% | 43.4% |
| Reddy [42] | 38 28 | 32 22 | 70 50 | 45.7% 44.0% | 54.3% 56.0% |
| Reveal [43] | 47 | 53 | 100 | 53.0% | 47.0% |
| Schwarzkopf [37]~ | -- | -- | -- | 28.6% | 71.4% |
| Tsuruoka [46] | 16 16 | 14 14 | 30 30 | 46.7% 46.7% | 53.3% 53.3% |
| Woldemariam [47]+ | 83 | 78 | 161 | 48.4% | 51.6% |

**KEY**
*170 participants in study but footwear measured in only 78 of these participants.
~71.4% refers to all three sub-cohorts, only one of which is the diabetes clinic.
+Male cases 28 + controls 55 = 83. Therefore 161 – 83 = 78 female participants.
-- = Unreported.
